# Preschool Staff Spot Social Communication Difficulties, But Not Restricted and Repetitive Behaviors in Young Autistic Children

**DOI:** 10.1007/s10803-018-03867-0

**Published:** 2019-02-04

**Authors:** Elisabeth Nilsson Jobs, Sven Bölte, Terje Falck-Ytter

**Affiliations:** 1 Uppsala Child and Baby Lab, Department of Psychology, Uppsala University, 751 42 Uppsala, Sweden; 2 Center of Neurodevelopmental Disorders (KIND), Division of Neuropsychiatry, Department of Women’s & Children’s Health, Karolinska Institutet, Stockholm, Sweden; 3 Center of Psychiatry Research, Stockholm County Council, Stockholm, Sweden; 4 Child and Adolescent Psychiatry, Stockholm County Council, Stockholm, Sweden; 5 Swedish Collegium for Advanced Study (SCAS), Thunbergsvägen 2, 752 38 Uppsala, Sweden

**Keywords:** Autism, Autism Spectrum Disorder, SRS-2, Preschool, Teacher informants, Child development

## Abstract

To fulfill the criteria for autism spectrum disorder (ASD), symptoms must be present across domains and contexts. We assessed preschool staff’s ratings of social communication and interaction (SCI) and restricted and repetitive behaviors (RRBs) in 3-year-old siblings of children with ASD, either diagnosed (n = 12) or not diagnosed (n = 36) with ASD, and typically developing siblings with no family history of ASD (n = 16). Ratings of SCI were more accurate than RRBs in differentiating the ASD group from the two other groups, and only the SCI ratings correlated with clinical assessment of social behavior. We conclude that while preschool staff ratings of SCI behaviors are adequate, ratings of RRBs should be treated with more caution.

Autism spectrum disorder (ASD) is a neurodevelopmental disorder and behaviorally characterized by difficulties in social communication and interaction (SCI) and restricted and repetitive behaviors (RRBs; Guthrie et al. 2013; Mandy et al. 2012; American Psychiatric Association 2013). Difficulties in SCI should be present in multiple contexts to fulfill diagnostic criteria. Since the clinical setting represents only one setting, it is crucial to get information about the child’s behavior from the parents. In cases when parents do not report the presence of autistic symptoms (Kim and Lord 2012; Zwaigenbaum et al. 2016) other informants, such as preschool staff, could contribute this information. Many young children in Europe attend preschool. In Sweden, 89% of all 2-year-olds and over 93% of all 3 to 5-year-olds attend preschool (nces.ed.gov;ec. europa.eu/eurostat). The preschool setting gives many opportunities to observe the child in interaction with peers during different activities (Westman Andersson et al. 2013; Huerta and Lord 2012). Clinicians sometimes visit the preschool as part of clinical assessment. Preschool visits are rather time-consuming and an alternative could be to collect information about autistic symptoms in the child from preschool staff. Preschool staff typically meet many children and may therefore rate behaviors more accurately than other informants (Branson et al. 2008). Supporting this view, research on the Social Responsiveness Scale (SRS; Constantino and Gruber 2005) has shown that teachers rate autistic symptoms more in line with clinical assessment by the Autism Observation Schedule (ADOS; Lord et al. 1999) than parents, for both school-aged children and toddlers (Duvekot et al. 2015; Azad et al. 2016). Correlations (Pearson’s *r*) between teacher ratings on the SRS and clinical assessment of autistic symptoms have been found to be between 0.31 and 0.42 (Constantino et al. 2007; Schanding et al. 2012; Reszka et al. 2013). However, these findings are based on total SRS scores, including both SCI and RRBs. To our knowledge, there is no data available showing what kind of ASD-related behaviors (i.e., difficulties of SCI and/or RRBs) that can be identified by teachers and preschool staff in children younger than 4 years of age.

The objective of this study was to investigate how accurate preschool staff are at reporting difficulties in SCI and RRBs in young children. We evaluated this both from a categorical (differentiating between groups; aim 1) and from a dimensional perspective (association between ratings and level of clinical autistic symptoms; aim 2). Specifically, the first aim was to investigate if preschool ratings of SCI and RRBs would differentiate children with and without ASD. We compared children with ASD, children with typical development (TD) and children with no ASD but with an older brother or sister with ASD. We expected that ratings on both domains would contribute uniquely to the differentiation between the groups. The second aim was to investigate if there was a specific association between preschool ratings and clinical assessment within each ASD-domain. That is, we investigated if there was an association between preschool ratings of SCI and clinical best practice assessment of social behavior (ADOS-2) on the one hand and between preschool ratings of RRB symptoms and clinical best practice assessment on RRBs (ADOS-2) on the other. We expected a positive correlation between preschool SCI and RRB ratings and the corresponding clinical measures of autistic symptoms.

## Methods

### Participants

The project was approved by the Regional Ethical Board in Stockholm. Participants were part of the longitudinal Early Autism Sweden (EASE; smasyskon.se) sibling project, including siblings with high and low risk for ASD. The project follows children from 5 months to 6 years of age. This study focused on the 3-year-olds in the project. High-risk siblings have an older sister or brother with ASD. About 18–20% of the high-risk-for-ASD group receive an ASD diagnosis, which is a higher percentage than in the general population, where rates are closer to 1–2% (CDC 2018; Messinger et al. 2013; Idring et al. 2015; Ozonoff et al. 2011). Even if these high-risk-for-ASD siblings will not receive an ASD diagnosis, many have subclinical autistic symptoms and/or other behavioral problems (Charman et al. 2017; Kantzer et al. 2018; Ozonoff et al. 2014; Landa et al. 2013; Bussu et al. 2018; Messinger et al. 2013). When evaluating how well a certain test or instrument identifies children with ASD, the sibling approach gives an opportunity to evaluate the measures’ ability not only to differentiate between groups with ASD and those with TD but also between groups of children that both have a familial history of ASD and different degrees of autistic symptoms.

The high-risk children were recruited through advertisement, the project’s website or at clinical units. For inclusion, these siblings should have at least one older sister or brother diagnosed with ASD. The low-risk children were recruited from a database of families, expressing an interest for participating in research projects. For inclusion, the low-risk children should have at least one older brother or sister with typical development and no first-hand relatives with known or suspected ASD. Exclusion criteria for both groups were pre-term birth (< 36 weeks) and confirmed or suspected medical problems.

Out of the 91 children that were assessed at 36 months during the data inclusion phase for the current study, 73 preschool ratings were obtained. Five of these were incomplete. After removal of another four participants (due to one control child having ASD; one child failing to fulfil initial inclusion criteria (detected retrospectively); no available diagnostic decision in one case; and one participant being a statistical outlier, see Analyses), preschool ratings for 64 children remained. The sample consisted of 36 (20 girls) children with high-risk-for-ASD with no diagnosis (HR-noASD); 12 (6 girls) with high-risk-for-ASD with ASD diagnosis (HR-ASD); and 16 (7 girls) with low risk for ASD controls (LR). All participants attended regular preschool, except for one participant in the HR-ASD group, attending a special education school.

Participant characteristics are presented in Table 1. There were no group differences in age. ADOS-2 assessments showed a pattern of HR-ASD > HR-noASD = LR for autistic symptoms. IQ and adaptive functioning showed a pattern of HR-ASD < HR-noASD = LR. Elevated autistic symptoms, signs of ADHD, speech and language impairment and developmental delay are also presented in Table 1. Elevated autistic symptoms were defined as comparison scores > 3 for the ADOS-2 Social Affect domain and raw scores > 1 for the ADOS-2 RRB scale. Speech and language impairment (SLI) was defined as a T-score ≤ 35 on the Expressive and/or Receptive scale of the Mullen Scales of Early Learning (MSEL). Developmental delay was defined as the MSEL Total composite IQ ≤ 70. ADHD symptoms in the child were evaluated by observations throughout the 36-month assessment day by an experienced clinician (see the Procedure section). Two participants in the LR group and 11 participants in the HR-noASD group had elevated autistic symptoms (in some cases combined with symptoms of SLI or ADHD). No participant in the LR group had SLI or ADHD symptoms. Thirteen participants in the HR-noASD group had either symptoms of SLI or ADHD. In the HR-ASD group, apart from elevated autistic symptoms in all participants, five participants had symptoms of either SLI or ADHD and one of these participants also had developmental delay.

**Table 1:** Participant characteristics

	LR n = 16			HR-noASD n = 36			HR-ASD n = 12			Group comparisons		
	M	SD	Range	M	SD	Range	M	SD	Range	*F*	*p*	Bonferroni post hoc
Age, months	36.64	1.05	4.93	37.19	1.32	5.36	37.74	2.53	8.97	1.73	0.186	*ns*
ADOS-2 CSs	2.56	1.26	4	3.31	1.65	7	7.25	1.77	6	34.88	< .001	HR-ASD > LR^3^; HR-ASD > HR-noASD^3^; LR = HR-noASD
ADOS-2 SA CSs	2.81	1.76	5	3.47	1.72	6	7.75	1.96	6	31.92	< .001	HR-ASD > LR^3^; HR-ASD > HR-noASD^3^; LR = HR-noASD
ADOS-2 RRB ASs	1.56	0.96	3	2.03	1.06	4	3.25	1.11	3	9.63	< .001	HR-ASD > LR^3^; HR-ASD > HR-noASD^2^; LR = HR-noASD
MSEL tot IQ	115.06	13.68	68	107.28	14.80	71	88.50	19.26	72	10.57	< .001	HR-ASD < LR^3^: HR-ASD < HR-noASD^2^; LR = HR-noASD
VABScomp	98.19	10.23	40	93.28	7.33	31	81.45	11.95	44	11.57	< .001	HR-ASD < LR^3^; HR-ASD < HR-noASD^3^; LR = HR-noASD

	n			n			n					
Tot elevated autistic and SLI/ADHD symptoms	2			19			12					
(Elevated autistic; SLI/ADHD)	(2; 0)			(11; 13)			(12; 5^a^)					

*LR* Low-Risk siblings; *HR-noASD* High-Risk siblings with no ASD; *HR-ASD* High-Risk siblings with ASD; *ADOS-2* Autism Observation Schedule-2; *CSs* Comparison Scores; *ASs* Algorithm Scores; *SA* Social Affect; *RRB* Repetitive and Restricted Behavior; *MSEL tot IQ* Mullen Scales of Early Learning, total scale; *VABScomp* Vineland Adaptive Scales, composite standard score; *SLI* Speech and Language Impairment; *ADHD* Attention Deficit Hyperactivity Disorder

^†^*p* ≤ .1; ^1^*p* ≤ .05; ^2^*p* ≤ .01; ^3^*p* ≤ .001

^a^One participant had developmental delay in this group

#### Preschool Ratings of Autistic Traits and Symptoms

We used the Social Responsiveness Scale-second edition (SRS-2; Constantino and Gruber 2012), which is a measure of autism as a continous trait that maps onto the DSM-5 SCI and RRB domains for ASD (American Psychiatric Association 2013; Frazier et al. 2014). The SRS-2 can be completed by either parents or teachers. It has four different forms depending on age, consisting of a total of 65 items, rated from 0 (not true) to 3 (almost always true). The Social Communication and Interaction subscale (SRS-2 SCI) is calculated from 53 items covering social awareness, social cognition, social communication and social motivation. The RRB subscale (SRS-2 RRB; previously called the Autistic Mannerism subscale) consists of 12 items. In this study, a research translation of the SRS-2 preschool form 2.5–4.5 years was used.

In addition, we administred the Repetitive Behavior Scale-Revised (Bodfish et al. 2000), which consists of 43 items, rated from 0 to 3 depending on behavior severity, covering the areas of stereotyped behavior, self-injurious behavior, compulsive behavior, routine behavior, sameness behavior and restricted behavior. Results can be summarized as a total score or number of items where it has been indicated that RRBs are present. Total raw scores were used in the current study.

### Measures

In this study, The DSM-5 SCI domain was operationalised and measured by preschool ratings on the SRS-2 SCI subscale. The DSM-5 RRB domain was operationalised and measured by preschool ratings on the RBS-R and the SRS-2 RRB subscale. The SRS-2 RRB subscale was included for comparison with the more comprehensive RBS-R scale, as the SRS-RRB subscale psychometrically coheres with the SRS-2 SCI subscale.

#### Ratings of Clinical Autistic Symptoms

The Autism Diagnostic Observation Schedule-2 (ADOS-2; Lord et al. 2012) is a semi-structured standardized clinical assessment of communication, social interaction, play and restricted and repetitive behavior. The ADOS-2 is comprised of two subscales (i.e., the Social Affect (SA) and the RRB subscales), which are consistent with the DSM-5 SCI- and RRB domains (American Psychiatric Association 2013). The total scores can either be presented as a raw score from 0 to 28 (termed “algorithm score”) or as standardized comparison scores (CSs) from 1 to 10; the latter allows comparisons between different ADOS-2 modules (in the current case, module 1 and 2; Lord et al. 2012). Separate algorithm scores and CSs can be calculated for the two ADOS-2 subscales Social Affect (SA) and RRB (Hus et al. 2014). In this study, CSs were used for the SA subscale. However, we chose algorithm scores for the ADOS-2 RRB subscale, as the Hus et al. (2014) standardization for RRB CSs do not convert raw scores into a continuous scale (i.e., the RRB CSs includes CSs 1 and 5–10 but not 2–4).

#### Developmental Level (IQ)

The Mullen Scales of Early Learning (MSEL; Mullen 1995) is a standardized developmental measure used to assess cognitive functioning between birth and 68 months. Composite scores are obtained from subscales on fine motor ability, visual reception and expressive and receptive language. The scales are presented in T-scores and a total composite standard score as a proxy for IQ.

#### Adaptive Functioning

The Vineland Adaptive Behavior Scales-2 (VABS-II; Sparrow et al. 2005) is a semi-structured parent-report questionnaire covering four different areas: Communication, Daily Living Skills, Socialization and Motor Abilities. Results are presented as an overall Adaptive Behavior Composite (ABC) in standard scores (mean = 100, SD = 15).

### Procedure

Data collection was conducted between March 2014 and June 2017. All assessments took place within the current sibling project during one day in a clinical setting and as close to 36 months as possible (Mean months for assessment as shown in Table 1: LR group = 36.6; HR-noASD group 37.2; HR-ASD group 37.7). All participants underwent diagnostic assessment, which was based on consensus of two experienced clinicians according to DSM-5 criteria (American Psychiatric Association 2013), using information from the ADOS − 2 module 1 (n = 8) or 2 (n = 56) and the Autism Diagnostic Interview-Revised (ADI-R; Rutter et al. 2003, 2008); the Vineland Adaptive Behavior Scales-2 (VABS-II; Sparrow et al. 2005; McDonald 2014; Mouga 2014) and the Mullen Scales of Early Learning (MSEL; Mullen 1995). Signs of ADHD symptoms in the child were observed throughout the day, both during formal assessment and during breaks in interactions with the parent. One of the two clinicians had contact with the family but was blind to the preschool ratings at the time of diagnostic assessment.

The SRS-2 and RBS-R were distributed to preschool through the parents and were sent back directly to the research team by regular mail or returned via the parents at the assessment day. The preschool ratings were completed about 1–4 weeks before the 36-months assessment day. At the time of rating, the preschool informants were blind to the result of the diagnostic assessment.

The preschool informants consisted of 36 preschool teachers with post-secondary education, 23 childcare providers with upper secondary high school training and five informants with other education or unknown educational history. The staff had known the child more than six months in 53 cases, less than six months in nine cases. In two cases, this information was missing.

### Analyses

Statistics were performed in SPSS 24 (IBMCorp 2016). Percentile confidence intervals 95% were based on 1000 bootstrap samples. Prior to regression analysis, one participant was considered as an outlier based on extreme values for Mahalanobis distances (> 11) and removed from the sample.

#### Differentiation Between Groups (Aim 1)

Group comparisons were conducted list-wise with one-way ANOVAs for the SRS-2 SCI subscale and the RBS-R. Results were analysed with Brown Forsyth F*. Corrections of *p*-values for multiple comparisons were calculated by the method of False discovery rate (Benjamini and Hochberg 1995). Post hoc results were reported according to Games Howell’s test. A two-tailed alpha-level of 5% was applied for significance.

Multinomial logistic regression was conducted with LR, HR-noASD and HR-ASD as categorical outcome variables with the HR-ASD group as reference category and the SRS-2 SCI subscale and RBS-R entered as predictor variables. Prior to analyses, these predictor variables were tested for linearity of the logit (binary regression with the predictor variables analysed separately with the groups LR vs HR-noASD; LR vs HR-ASD; HR-noASD vs HR-ASD as outcome variables). The interactions had values greater than 0.05 (*p*s ≥ .098), indicating no violation of this assumption.

#### Relations Between the ADOS-2 and the Preschool Rating Scales (Aim 2)

Pearson’s *r* bivariate correlations between the ADOS-2 SA and ADOS-2 RRB subscales and the variables SRS-2 SCI and RBS-R were conducted. Multiple linear regressions were conducted with the ADOS-2 SA CSs entered as the outcome variable and both the SRS-2 SCI and the RBS-R scores entered simultaneously as predictor variables in the one and the same block. The corresponding analysis was conducted with the ADOS-2 RRB algorithm scores as outcome variable. Prior to the regression, normality of residuals and homoscedasticity were visually inspected and found not to violate assumptions. Here we focused on the high-risk-for-ASD children (i.e., the HR-no ASD and HR-ASD together), excluding the LR group. We chose to focus on the two HR groups as these both come from a similar familial background and have a large variation in elevated autistic symptoms and signs of ADHD/SLI.

## Results

### Differentiation Between Groups (Aim 1)

Group comparisons for the SRS-2 SCI subscale and the RBS-R are presented in Table 2. The ANOVAs showed a significant difference between all three groups on the SRS-2 SCI subscale. There was no significant group difference on the RBS-R scale, however post hoc analyses showed a trend of a HR > LR pattern.

**Table 2:** Group comparisons, SRS-2 and RBS-R

	LR n = 16			HR-noASD n = 36			HR-ASD n = 12			Group comparisons		
	M	SD	Range	M	SD	Range	M	SD	Range	*F* Brown-Forsythe (df2)	Corrected *p*	Post hoc Games-Howell
SRS-2tot	15.06	10.85	39	27.94	17.49	73	50.83	27.27	81	10.73 (19.95)	.001	HR-ASD > LR^2^; HR-ASD > HRnoASD^1^; HRnoASD > LR^2^
SRS-2 SCI	14.19	10.17	37	25.06	14.75	63	46.08	24.46	74	10.78 (19.60)	.001	HR-ASD > LR^2^; HR-ASD > HRnoASD^1^; HRnoASD > LR^2^
RBS-R	0.50	0.89	3	2.25	3.98	16	4.50	5.62	15	3.31 (17.97)	.060†	HR-ASD > LR^†^; HRnoASD > LR^1^; HR-ASD = HR-noASD

*LR* Low-Risk siblings; *HR-noASD* High-Risk siblings with no ASD; *HR-ASD* High-Risk siblings with ASD; *SRS-2tot* Social Responsiveness Scale-2, total scores; *SCI* Social Communication and Interaction; *RBS-R* Repetitive Behavior Scale-Revised

^†^*p* ≤ .1; ^1^*p* ≤ .05; ^2^*p* ≤ .01; ^3^*p* ≤ .001

The results for the logistic multinomial regression analyses of the SRS-2 SCI subscale and the RBS-R are shown in Table 3. Only the SRS-2 SCI subscale contributed uniquely to the differentiation between the HR-ASD and the LR groups as well as between the HR-ASD and HR-noASD groups.

**Table 3:** Multinomial logistic regression, SRS-2 SCI and RBS-R

Group:	*b* (SE)	95% CI for odds ratio		
		Lower	Odds ratio	Upper
LR vs HR-ASD				
Intercept	3.66 (1.02)^3^			
SRS-2 SCI	− 0.13 (0.04)^2^	0.81	0.88	0.96
RBS-R	− 0.14 (0.28)	0.50	0.87	1.52
HRnoASD vs HR-ASD				
Intercept	3.08 (0.85)^3^			
SRS-2 SCI	− 0.06 (0.03)^2^	0.90	0.94	0.99
RBS-R	0.03 (0.09)	0.86	1.03	1.23

*LR* Low-Risk siblings; *HR-noASD* High-Risk siblings with no ASD; *HR-ASD* High-Risk siblings with ASD; *SRS-2 SCI* Social Responsiveness Scale, Social Communication and Interaction; *RBS-R* Repetitive Behavior Scale-Revised; R^2^ = 0.31 (Cox & Snell); 0.35 (Nagelkerke); Model X^2^ (4) = 23.28

Reference: HR-ASD group

^†^*p* ≤ .1; ^1^*p* ≤ .05; ^2^*p* ≤ .01; ^3^*p* ≤ .001

### Relations Between the ADOS-2 and the Preschool Rating Scales (Aim 2)

As previously pointed out, the dimensional analysis was based on the HR group only. The correlations between ADOS-2 Social Affect CSs, ADOS-2 RRB- and SRS-2 SCI as well as the RBS-R are presented in Table 4. The SRS-2 SCI ratings were positively correlated with the ADOS-2 Social Affect CSs but not the ADOS-2 RRB algorithm scores. The RBS-R was not correlated with any of the two ADOS-2 subscales.

**Table 4:** Pearson’s *r*, HR group, n = 48

	1	2	3
1 ADOS-2 SA CSs	–		
2 ADOS-2 RRB ASs	0.462^3^	–	
3 SRS-2 SCI	0.504^3^	0.108	–
4 RBS-R	0.136	0.077	0.564^3^

*ADOS-2* Autism Observation Schedule-2; *SA* Social Affect; *CSs* Comparison Scores; *ASs* Algorithm Scores; *RRB* Repetitive and Restricted Behavior; *SRS-2 SCI* Social Responsiveness Scale, Social Communication and Interaction, *RBS-R* Repetitive Behavior Scale-Revised

^†^*p* ≤ .1; ^1^*p* ≤ .05; ^2^*p* ≤ .01; ^3^*p* ≤ .001

Multiple linear regression with the ADOS-2 SA CSs as outcome variable and the SRS-2 SCI subscale and the RBS-R as predictor variables resulted in a significant overall model (F (2,45) = 9.01, *p* = 0.001, R^2^ = 0.286; Adj R^2^ = 0.254), with the SRS-2 SCI explaining 27% of the variation in ADOS-2 SA CSs. The RBS-R scale did not contribute significantly to the model (*b* = − 0.124 [β = − 0.217], *p* = 0.162).

Multiple linear regression with the ADOS-2 RRB scale as outcome variable and the SRS-2 SCI and RBS-R as predictor variables did not result in a significant overall model (F(2,45) = 0.274, *p* = 0.762, R^2^ = 0.012; Adj R^2^ = − 0.032).

### Comparative Analyses on RRB-Scales

In the additional analyses for the SRS-2 RRB subscale, the results were in line with the results for the RBS-R, with the ANOVA showing a HR > LR pattern for group means. Furthermore, no significant correlations were found between the SRS-2 RRB subscale and the ADOS-2 RRB subscale in the HR sample and the SRS-2 RRB showed no significant prediction on either group or ADOS-2 RRB scores.

## Discussion

In this study, we investigated if preschool staff could differentiate HR children with ASD from HR children without ASD and LR children by ratings on SCI behaviors (SRS-2 SCI subscale) and RRBs (RBS-R scale). Moreover, we investigated the relation between preschool staff ratings on the SRS-2 SCI subscale and the RBS-R and clinical assessment of autistic symptoms on the ADOS-2 SA and RRB subscales in the HR group. In line with our expectations, we found that preschool staff differentiated between all three groups (LR, HR-ASD and HR-noASD) on SCI behaviors and that the SCI ratings were significantly associated with clinical assessment on the ADOS-2 SA CSs. Our results are in line with earlier research findings that preschool teachers can differentiate social deficits in children with ASD from those with TD (Major et al. 2017) and that teacher ratings are closely related to clinical assessment (Duvekot et al. 2015; Azad et al. 2016). However, against our expectations, ratings on the RBS-R scale showed no differentiation between the HR groups. Moreover, the RBS-R did not correlate with the ADOS-2 RRB subscale.

Preschool ratings on the SRS-2 SCI subscale contributed uniquely to group differentiation. In contrast, no unique variance was captured by the RRB scale (Table 3). For the mean group comparisons (Tables 1, 2), preschool ratings on the SRS-2 SCI subscale differentiated the LR group from the HR groups. In contrast, the clinical assessment on the ADOS-2 SA subscale differentiated between the HR-ASD group from the groups with no ASD. One explanation for this difference in profiles could be that the preschool staff may have had knowledge of the children with familial risk of ASD (i.e., being part of the HR group) and as a result, over-rated the children in the HR-noASD group. Another possible explanation could be that the SRS-2 SCI subscale picks up on other symptoms not directly related to ASD, but nevertheless affects the distinction between LR and HR children. The SRS-2 SCI subscale has been found to be elevated in the presence of ADHD traits and speech and language impairment can be mistaken for autistic symptoms in many cases (Factor et al. 2017; Garrido et al. 2017; Janvier et al. 2016). As there were comorbid symptoms of ADHD or speech and language impairment in both HR groups (5 out of 12 in the HR-ASD group and 13 out of 36 in the HR-noASD group), these symptoms may have influenced results. In contrast to the SCI domain, the RRB domain was found to be more difficult to evaluate for the preschool staff. RRBs include repetitive motor and speech behaviors, sensory interests or aversion and insistence on sameness manifested by rigidity, routines, and restricted interests (Uljarević et al. 2017; Frazier et al. 2014; Bishop et al. 2013). RRBs are present in children with TD as well as in children with other developmental delays. Children with ASD often have a greater range of behaviors or they are expressed with higher intensity than in other groups, but this can still be hard to evaluate (Harrop et al. 2013; Joseph et al. 2013). Moreover, both type and frequency can be very similar in HR-groups with and without ASD (Damiano et al. 2013). Thus, while the clinicians could differentiate the ASD-group from the other groups on RRBs by the ADOS-2, these differences may be too subtle for non-clinicians. Preschool staff are probably more used to identifying atypical social behavior than RRBs. Another explanation for the difference in results could be that unlike the ADOS-2 RRB subscale, the RBS-R does not include ratings on repetitive and idiosyncratic speech. Potentially, adding items on stereotypic speech could lead to clearer differentiation between groups. Nevertheless, the group mean comparisons showed that preschool staff did differentiate the LR group from the HR-noASD group, indicating that some difference was spotted between the groups. As the expert clinical assessment on the ADOS-2 indicated no difference between the LR and HR-noASD groups on RRBs, this may represent differences other than RRBs as previously discussed for the SCI behaviors. Indeed, it has been found that parent ratings on the RBS-R significantly correlate with ADHD symptoms (Gabriels et al. 2005). Given that 10 children in the HR-noASD groups had ADHD-symptoms, the difference spotted by preschool staff between the groups could be due to these symptoms.

### Limitations

One evident limitation in our study is the small sample size. Nevertheless, the main conclusion from the study is that preschool staff are more accurate in their reporting of SCI than of RRBs. Another limitation is that we do not know to what extent our result can be generalized to preschool informants in other cultures. Another limitation is that we did not have information whether the preschool staff new about the child’s group status (HR vs LR).

Critically however, the clinicians were not aware of the results of the ratings at the time of the diagnostic evaluation, and the preschool raters were unaware of the diagnostic status of the child at the time of their assessment. Thus, in this important respect, the study was double blind. Finally, we compared the SRS-2 SCI subscale, which is a population focused scale with the RBS-R, which is a scale directed to a clinical group. However, as noted, a highly similar pattern of results was found on the RRB subscale of the SRS-2, suggesting that our findings are not linked to the use of a particular RRB operationalization.

### Clinical Implications and Future Directions

This is the first study that evaluates preschool staff’s report on the specific RRB domain within ASD. The contrast between the clear differentiation between groups on SCI behaviors and the unclear result for RRBs suggest that preschool staff may find it easier to assess social behaviors that are absent, than to identify and differentiate RRBs that are present. For example, it may be easier to notice that a child does not respond to his or her name during morning assembly than to spot that a child engages in finger mannerisms. Not taking part in social activity may also have a greater impact on group dynamics than if a child plays in a repetitive way. The difference in results, indicate that more education for preschool staff may be needed regarding the features of RRBs. Further research on the value of educational efforts for preschool staff on RRBs is needed in order to evaluate if it can increase identification and differentiation between groups. An important clinical implication of our result is that total scale scores on ratings of autistic symptoms from preschool staff should be evaluated with caution and rather be evaluated by separate subscales on the SCI and RRB domains when possible.

## Conclusion

Ratings on SCI behaviors by preschool staff are in line with clinical assessment and can be a reliable source in differentiating children with ASD from those who have not. However, this is not the case for ratings on RRBs. Before having evaluated educational effects on the discrimination of RRBs, our findings indicate that direct observation of the child by the clinician is recommended, especially in regard to RRBs, rather than using preschool staff exclusively as informants in the preschool context.
